# Sialodochitis fibrinosa (kussmaul disease) report of 3 cases and literature review

**DOI:** 10.1097/MD.0000000000005132

**Published:** 2016-10-21

**Authors:** Bryan Josue Flores Robles, Beatriz Brea Álvarez, Abel Alejandro Sanabria Sanchinel, Robert Francis Andrus, María Espinosa Malpartida, Consuelo Ramos Giráldez, Ana Lerma Verdejo, Carolina Merino Argumanez, Jose Antonio Pérez Pimiento, Camen Bellas Menéndez, Luis Fernando Villa Alcázar, José Luis Andréu Sánchez, Mercedes Jiménez Palop, Hildegarda Godoy Tundidor, José Campos Esteban, Jesús Sanz Sanz, Carmen Barbadillo Mateos, Carlos María Isasi Zaragoza, Juan Bartolomé Mulero Mendoza

**Affiliations:** a Rheumatology Division; b Radiodiagnostic Division, Puerta de Hierro Hospital, Madrid; c Neurology Division, Lozano Blesa Hospital, Zaragoza, Spain; d Independence Polyclinic, Belize; e Haematology Division; f Allergology Division; g Anatomical Pathology Division, Puerta de Hierro Hospital, Madrid, Spain.

**Keywords:** allergic parotitis, Fibrinous sialodochitis, Kussmaul disease, sialodochitis fibrinosa

## Abstract

**Background:**

Sialodochitis fibrinosa is a rare disease which is characterized by recurring episodes of pain and swelling of the salivary glands due to the formation of mucofibrinous plugs. Analytic studies ascertain elevated levels of eosinophils and immunoglobulin E (IgE). Imaging studies such as magnetic resonance imaging (MRI) and sialography reveal dilation of the main salivary duct (duct ectasia). Treatment is initially supportive, consisting of compressive massages, and use of antihistamines and/or corticosteroids.

**Material and methods:**

In the following, 3 cases of sialodochitis fibrinosa are presented which were diagnosed in a third level hospital during the period of 2008 and 2016, as well as a literature review of all cases reported to our knowledge.

**Results:**

Of the 41 cases found, including the 3 of this article, 66% were women with an average age of 45 years old. However, 75% of reported cases were of Japanese heritage. Involvement of the parotid glands was more frequent than the submandibular glands. In more than half of all cases treatment with compressive massages, antihistamines and/or corticosteroids was effective.

**Conclusion:**

Clinicians should consider sialodochitis fibrinosa as a diagnostic possibility when presented with cases of recurring parotid and submandibular gland tumescence.

## 1 Introduction

Sialodochitis fibrinosa was first described in 1879 by Kussmaul^[1]^ as pain and diffuse swelling of the parotid and/or submandibular glands caused by the obstruction of the salivary ducts by mucofibrinous plugs. Diagnosis is difficult and may be confused with other clinical entities such as Sjögren syndrome or infectious sialadenitis.^[2]^ Although diagnostic criteria has not been defined, the typical findings of described cases are: (a) recurring episodes of parotid/submandibular gland swelling, (b) discharge of mucofibrinous plugs with a high content of eosinophils, (c) elevated levels of IgE and/or eosinophils in blood, (d) presence of concomitant allergic processes such as asthma or allergic rhinitis, (e) irregular dilation of the main salivary ducts as seen by imaging studies (sialography or MRI), (f) glandular biopsy showing lymphocytic infiltrates and abundant eosinophils within the interstium surrounding the salivary ducts.^[3,4]^ The etiology of this disease remains unknown although the most accepted hypothesis is that it is an allergic process.^[4–6]^ Treatment varies according to the severity of the disease, nevertheless initial supportive treatment with massages and hydration is recommended in conjuncture with the use of antihistamines and/or corticosteroids. In refractory cases, corticosteroid infiltration and dilation of the parotid duct has been done.^[3]^ The following presents 3 cases of sialodochitis fibrinosa seen in a third-level hospital with a description of their clinical presentation, diagnostic workup, and management.

## 2 Method

Three patients with sialodochitis fibrinosa consulting in a third-level hospital were evaluated during the period of 2008 to 2016, of whom their sociodemographic characteristics, clinical presentations, study imaging findings, and cytological findings were described. Subsequently, a thorough literature review was done, finding 28 articles reporting a total of 41 cases including the 3 from this report. The search timeframe included articles from April 1879, the time of Kussmaul's initial description, until June 2016. Searching was done using the terms sialodochitis fibrinosa, fibrinous sialodochitis, Kussmaul disease, and allergic parotitis, within platforms such as Pubmed, Embase, Medline, Scopus, as well as nonindexed journals found in the hospital's virtual library and internet browser (mainly Japanese journals). Furthermore, the bibliography of each article was reviewed to capture more reported cases. In this review, all articles were taken into account regardless of the language in which they were written. The study was approved by the hospital ethics committee investigation (CEIC).

## 3 Case reports

### 3.1 Case 1

A 41-year-old Spaniard woman consulted because of recurring bilateral parotid swelling and pain during the past 4 years which were triggered by food consumption. Episodes initially occurred every 3 to 4 days and progressed until they occurred daily during the last weeks before consultation. After meals she would massage her parotid glands, relieving the pain and swelling as mucous secretions would be expressed into her buccal cavity. The only past medical history finding mentioned was allergic rhinitis. She was a nonsmoker with no history of alcohol consumption. She had no family history of allergies. She had no history of fever, weight loss, or other constitutional symptoms. Physical exam revealed bilateral parotid swelling with pain on palpitation and associated erythema. Intraoral examination showed extruded mucous plugs and secretion from the Stensen ducts upon compressing the glandular region. A sterile cotton swab was used to obtain a sample of the secretion for slide analysis.

**Figure 1:**
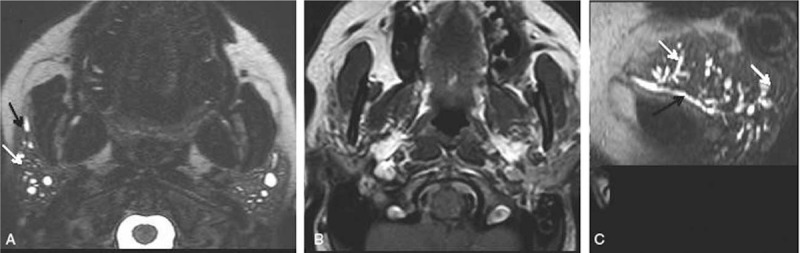
(A) MRI fat saturated T2-weighted axial plane. (B) MRI T1-weighted axial plane. (C) Oblique sagittal reconstruction. The parotid glands are slightly reduced in size and present multiple cystic dilations in the intraglandular ducts (white arrows) and mild dilation of main ducts (black arrows). MRI = magnetic resonance imaging.

**Figure 2:**
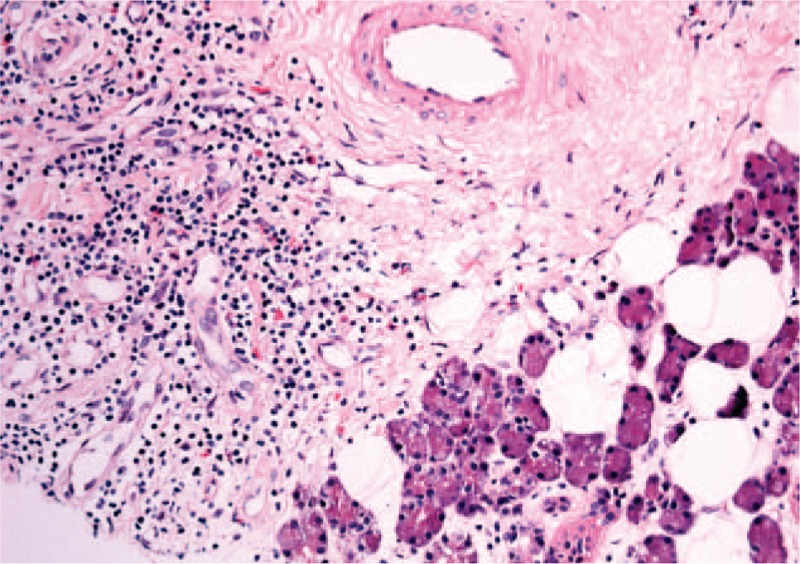
Interstitial filtrate of mature lymphocytes T and B and abundant eosinophils surrounding the salivary ducts.

Laboratory results showed a complete blood count (CBC) with a leukocyte count of 4970 μL, and eosinophil count of 570 μL, IgE levels of 93.5 KU/L (reference range 4.2–592), and C-reactive protein (CRP), erythrocyte sedimentation rate (ESR), rheumatoid factor (RF), anticitrullinated protein antibody (ACPA), angiotensin-converting enzyme (ACE), proteinogram and IgG4 with normal levels. Antinuclear antibodies (ANAs), antineutrophil cytoplasmic antibodies (ANCAs) were negative, serology for hepatitis B virus (HBV), hepatitis C virus (HCV), human immunodeficiency virus (HIV) were negative, Mantoux test was negative, and food allergy studies were negative. Chest and neck x-rays, neck tomography, salivary gland scintigraphy, 18F-FDG PET/CT were normal. An MRI of the parotid glands reported multiple cystic dilations of the intraglandular ducts and dilation of the main drainage duct (Fig. 1). The glandular secretion was stained demonstrating numerous eosinophils. Glandular biopsy revealed an interstitial filtrate of mature lymphocytes T and B (CD3+ and CD20+) and abundant eosinophils surrounding the salivary ducts (Fig. 2). The patient was diagnosed with sialodochitis fibrinosa.

Treatment was started with prednisone 10 mg/day and loratadine 10 mg/day. Accordingly, the patient showed good clinical response after the first week and then was found to be asymptomatic after 5 months. Six months after the diagnosis, the patient continued treatment with loratidine 10 mg/day and prednisone 2.5 mg/q.a.d.

### 3.2 Case 2

A 46-year-old Peruvian woman consulted because of recurring episodes of swelling and pain in both parotid regions during the past 5 years which were triggered by the consumption of various foods, although the patient did not specify the food types. Episodes were associated with the expulsion of mucous secretions as the patient would massage her parotid glands postprandially. She had a significant medical history of asthma and chronic rhinoconjunctivitis. She was a nonsmoker and had no history of alcohol consumption. She had no family history of allergies. Physical exam revealed bilateral parotid swelling which was more pronounced on the right side. Intraoral examination revealed extruded mucous secretion upon compressing the right parotid region.

**Figure 3:**
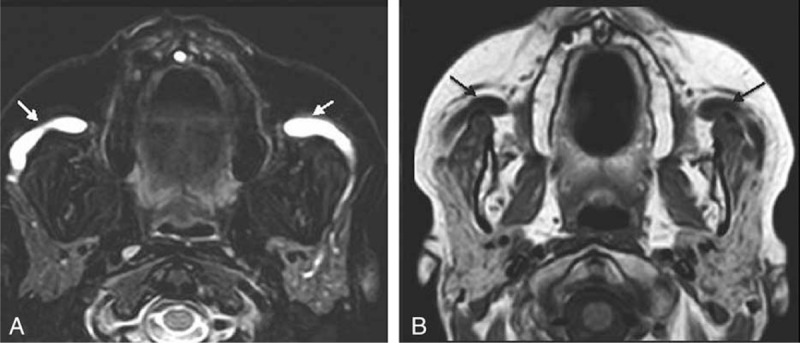
(A) MRI fat saturated T2-weighted axial plane. (B) MRI T1-weighted axial plane. The parotid glands have a conserved signal and size. There is no evidence of intaparotid solid or cystic lesions. There is significant dilation of the main drainage ducts (arrows). MRI = magnetic resonance imaging.

Laboratory results showed a CBC with a leukocyte count of 8290 μL and eosinophil count of 820 μL, and IgE level of 200 KU/L, and CRP, levels of PCR, ESR, RF, ACPA, ACE, proteinograma, and IgG4 with normal levels. ANAs and ANCAs were negative, serology for HVB, HVC, and VIH were negative, and the Mantoux test was negative. Among imaging studies, a chest x-ray was normal, whereas an MRI of the salivary glands demonstrated dilation of both main drainage parotid ducts (Fig. 3). Food allergy studies were negative. A stain of the salivary gland secretion presented abundant mixed-type inflammatory cellularity with a high proportion of eosinophils (20%) in a proteinaceous background. Parotid gland biopsy was refused by the patient. The patient controlled her symptoms with irregular use of antihistamines.

### 3.3 Case 3

A 28-years-old Spaniard woman consulted because of a 20-year history of recurring left-side parotid swelling. Episodes were not related with food consumption, yet the episodes were turning more frequent. Past medical history only included seasonal rhinitis. Physical exam presented left parotid swelling with expulsion of mucous material from the Stensen duct upon compressing the left glandular region, whereas the right-side parotid region presented no relevant physical exam findings. Laboratory results showed a CBC with a leukocyte count of 8590 μL and eosinophil count of 600 μL. The rest of laboratory results were normal. An MRI of the parotid glands showed both Stenon ducts to be dilated. A stain of the salivary gland secretion presented abundant eosinophils. The patient remained asymptomatic with periodic compressive massages.

## 4 Discussion

The swelling of the salivary glands is a relatively common symptom which can be caused by several different processes whether inflammatory or noninflammatory.^[4]^ Among the differential diagnosis of parotid swelling, it is important to rule out secondary and systemic causes, one of them being sialodochitis fibrinosa (also known as Kussmaul disease). Sialodochitis fibrinosa was first described in 1879, as salivary gland swelling, affecting mainly the parotid glands, habitually bilateral and recurring, with the expulsion of mucous secretion from the salivary ducts upon compressing the affected glandular regions, in patients with a past medical history of allergic processes and elevated eosinophils and IgE. Stained samples of the mucous material demonstrate abundant eosinophils. Imaging studies, such as MRI and sialography, demonstrate dilated salivary ducts and ectasia. Salivary gland biopsy shows lymphocytic infiltrates with abundant eosinophils in the periductal interstitium. Currently there are no established criteria for diagnosis being an extremely rare disease. Diagnosis is difficult and done by exclusion, requiring a high index of suspicion for its confirmation. The differential diagnosis of this disease includes Sjögren syndrome, sarcoidosis, lymphoma, IgG4-related disease, bacterial and viral infectious sialadenitis, sialolithiasis, among others.

Although the exactitude of the epidemiological and demographical characteristics is unknown, in this study it was found (Table 1) that 66% of the 41 reported cases were women with an average age of 45-years old, and the parotid glands was predominantly more affected than the submandibular glands (26 cases affecting the parotid glands and 15 cases affecting the submandibular glands). Almost 75% of the patients from reviewed cases were of Japanese heritage.

**Table 1:**
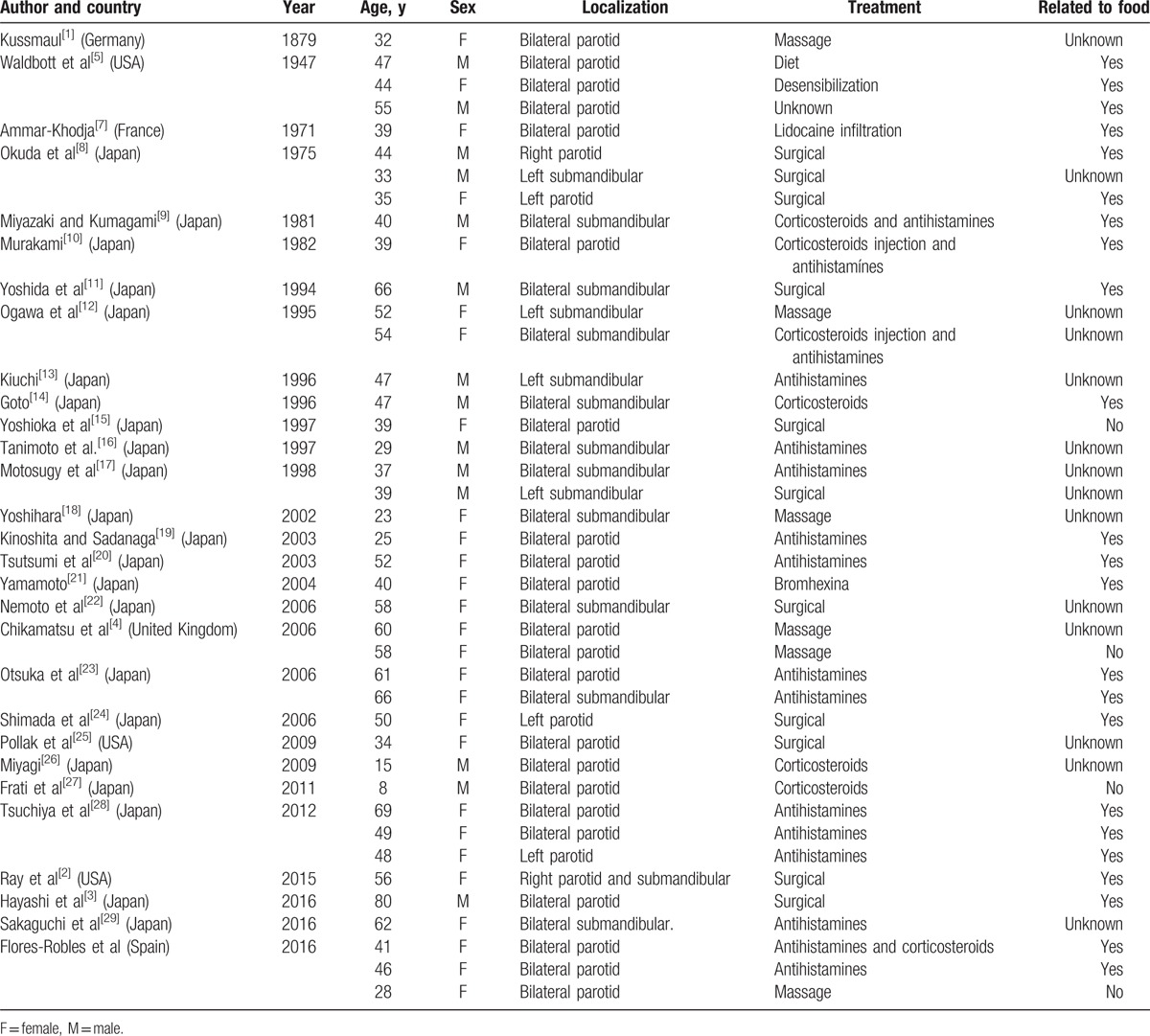
Case reports of sialodochitis fibrinosa (Kussmaul disease).

Although the etiology of sialodochitis fibrinosa remains unknown, it is suggested that it is caused by an allergic process related to the consumption of certain foods, namely tuna fish, wheat, lettuce, and legumes such as lima beans.^[5,6]^ In this study, it was noted that 23 case reports specifically referenced the presence of food-related exacerbations; 4 case reports established that the symptoms were not food related; and 14 case reports made no mention of food-related symptoms. It is, therefore, deduced that food allergies should be sought out in a majority of patients. Of the 23 case reports affirming food-related symptoms, the majority did not specify the food type.

The treatment of this disease depends on the severity of the symptoms. Generally, compressive massages of the salivary glands, abundant hydration, and the administration of antihistamines with or without corticosteroids should be considered as the initial management. In reviewing 41 cases, it was noted that more than half of the patients improved with only massaging and antihistamines. In 11 cases, an invasive procedure was required such as instrumental dilation of the parotid ducts and/or partial gland resection.^[3]^

Of the 3 case reports presented in this article, it was observed that 2 patients presented symptoms that were triggered by food consumption, although the specific food types were not determined. The patient mentioned in the first case presented good treatment response and achieved complete remission of her symptoms by taking low-dose corticosteroids together with an antihistamine.

Together with the series described by Okuda (1975)^[8]^ and Tsuchiya (2012),^[28]^ this is the largest series of sialodochitis fibrinosa described thus far; also, this is the first literature review to date written in English which delimits demographic characteristics, clinical presentations, and therapeutic management of this disease. Due to the limited number of sialodochitis fibrinosa cases reported, incomplete information regarding food-related symptoms, and incomplete recall of the specific food types, it is challenging at the present to provide meaningful conclusions on the association of this disease and food allergies. Nevertheless, this also indicates that further research is warranted.

In conclusion, patients undergoing diagnostic workup for episodes of recurring parotid or submandibular swelling, where systemic and infectious causes have been ruled out, sialodochitis fibrinosa should be considered as a potential cause. Sialodochitis fibrinosa is a disease which may be easily treated by conservative means, therefore improving the patients’ quality of life.
